# Impact of intensive care unit admission on cancer patients: enhancing long-term survival through better understanding

**DOI:** 10.62675/2965-2774.20240212-en

**Published:** 2024-10-31

**Authors:** Ana Paula Agnolon Praça, Antonio Paulo Nassar, Pedro Caruso

**Affiliations:** 1 Intensive Care Unit A.C. Camargo Cancer Center São Paulo SP Brazil Intensive Care Unit, A.C. Camargo Cancer Center - São Paulo (SP), Brazil.

By 2030, 22,300 million new cases of cancer will be diagnosed worldwide. Given these numbers, it is imperative to evaluate all clinical scenarios for patients with cancer, including those who are admitted to the intensive care unit (ICU) after a cancer diagnosis and discharged home. Many studies have evaluated short- and long-term outcomes and associated risk factors in patients with cancer who require planned or unplanned ICU admission. Most of these studies included all patients admitted to the ICU in their analyses. However, the subpopulation of patients admitted to the ICU who have recovered and were discharged home has not been adequately evaluated. This population needs to be evaluated, and the study by Puxty et al.^(1)^ is an important step in understanding the impact of ICU admission on long-term outcomes in patients with cancer.

In a retrospective study, Puxty and colleagues reported that patients with nonmetastatic solid tumors who were admitted to the ICU in the first two years after their cancer diagnosis had worse progression-free survival than patients who were hospitalized but did not require ICU admission. Using a national database, Van der Zee et al.^(2)^ reported that approximately 30% of patients with cancer who survived hospital admission died within 1 year. Gheerbrant et al.^(3)^ also used a national database and reported 40% 6-month mortality after ICU discharge, with the presence of metastases independently associated with lower survival. The two previous studies included only unplanned ICU admissions, whereas the study by Puxty et al.^(1)^ included planned and unplanned ICU admissions.

The findings of Puxty et al.^(1)^ are important and represent a major step toward a better understanding of patients with cancer discharged home after critical illness; however, two questions remain unanswered. The first question is as follows: Do all patients with cancer experience negative consequences from their ICU stay? The second question is as follows: What are the causes of the associations of ICU admission with cancer progression and decreased long-term survival?

Patients with planned ICU admission, few organ dysfunctions, and a short ICU length of stay are likely less affected by ICU admission, whereas patients with unplanned ICU admission, multiple organ dysfunctions, and a long ICU length of stay are likely affected and have worse progression-free survival than patients with cancer but no ICU admission. The study by Puxty et al.^(1)^ does not allow us to clearly identify which patients are negatively affected by ICU admission because the data did not allow for a detailed characterization of the patients admitted to the ICU. For example, we do not know the clinical severity of patients on admission or their clinical course during the ICU stay.

Two hypotheses may explain the associations of ICU admission with cancer progression and decreased long-term survival. The first hypothesis is interruption, delay, or modification of cancer treatment because changes in cancer treatment, even small delays, are associated with decreased survival. The second hypothesis is that prolonged immunosuppression after critical illness may lead to cancer progression. The association between critical illness and immunosuppression is well established, and it is possible that critical illness-induced immunosuppression may cause cancer progression due to the immunosuppressive signatures of critical illness in the tumor microenvironment, as recently summarized in sepsis, a common cause of ICU admission in patients with cancer.^(4)^ In support of the link between immunosuppression and cancer progression, a recent study revealed a one-year increased cancer incidence in sepsis survivors compared with the general population.^(5)^ If these two hypotheses are confirmed in future studies, or at least one of them, it will be possible to intervene to reduce cancer progression and increase survival in patients with cancer after ICU admission. One potential intervention is to avoid altering cancer treatment by raising awareness among oncologists to prescribe programmed anticancer treatment or an alternative appropriate for the patient's clinical condition during hospitalization and by providing clinical and socioeconomic support to avoid delay or interruption of cancer treatment after hospital discharge. If prolonged immunosuppression after critical illness is shown to be relevant to cancer progression, another intervention could be the addition of treatments that increase immune competence, such as immunotherapy.

In conclusion, Puxty and colleagues confirmed the association between ICU admission and poor progression-free survival in patients with solid tumors. Further studies are needed to identify which patients are adversely affected by ICU admission and to determine the causes linking ICU admission to cancer progression and lower mortality. Once the causes are known, we can implement interventions to reduce cancer progression and increase patient survival.

## References

[1] Puxty K, Keith R, McPeake J, Morrison D, Shaw M (2024). Rate of non-metastatic solid tumor progression following critical illness: a prospective cohort study of UK Biobank participants. Critical Care Science.

[2] van der Zee EN, Termorshuizen F, Benoit DD, de Keizer NF, Bakker J, Kompanje EJ (2022). One-year mortality of cancer patients with an unplanned ICU admission: a cohort analysis between 2008 and 2017 in The Netherlands. J Intensive Care Med.

[3] Gheerbrant H, Timsit JF, Terzi N, Ruckly S, Laramas M, Levra MG (2021). Factors associated with survival of patients with solid Cancer alive after intensive care unit discharge between 2005 and 2013. BMC Cancer.

[4] Mirouse A, Vigneron C, Llitjos JF, Chiche JD, Mira JP, Mokart D (2020). Sepsis and cancer: an interplay of friends and foes. Am J Respir Crit Care Med.

[5] Hästbacka J, But A, Strandberg G, Lipcsey M (2023). Risk of malignant disease in 1-year sepsis survivors, a registry-based nationwide follow-up study. Crit Care.

