# Supplementary material for: Impact of intensive care unit admission on cancer patients: enhancing long-term survival through better understanding
Source: Crit Care Sci. 2024 Oct 31;36:e20240212en. doi: 10.62675/2965-2774.20240212-en (PMC11634238; doi:10.62675/2965-2774.20240212-en)
Supplement: Translation

# Translation: Os efeitos da internação em unidades de terapia intensiva em pacientes com câncer: aumento da sobrevida em longo prazo por meio de um melhor entendimento

**DOI:** 10.62675/2965-2774.20240212-pt

**Authors:** Ana Paula Agnolon Praça, Antonio Paulo Nassar, Pedro Caruso

**Affiliations:** 1 São Paulo SP Brasil Unidade de Terapia Intensiva, A.C. Camargo Cancer Center - São Paulo (SP), Brasil.

Até 2030, 22,3 milhões de novos casos de câncer serão diagnosticados em todo o mundo. Diante desse número, é imperativo avaliar todos os cenários clínicos dos pacientes com câncer, inclusive aqueles internados na unidade de terapia intensiva (UTI) após o diagnóstico de câncer que recebem alta para casa. Muitos estudos avaliaram os desfechos em curto e longo prazo e os fatores de risco associados em pacientes com câncer que precisaram de internação planejada ou não planejada na UTI. A maioria desses estudos incluiu todos os pacientes admitidos na UTI em suas análises. No entanto, a subpopulação de pacientes internados na UTI que se recuperaram e receberam alta para casa não foi avaliada adequadamente. Essa população precisa ser avaliada, e o estudo de Puxty et al.^(1)^ é um passo importante para entender o efeito da internação na UTI nos desfechos em longo prazo de pacientes com câncer.

Em um estudo retrospectivo, Puxty et al.^(1)^ relataram que pacientes com tumores sólidos não metastáticos internados na UTI nos primeiros 2 anos após o diagnóstico de câncer tiveram um intervalo livre de progressão menos favorável do que pacientes hospitalizados sem necessidade de internação na UTI. Van der Zee et al.,^(2)^ valendo-se de um banco de dados nacional, relataram que aproximadamente 30% dos pacientes com câncer sobreviventes à internação hospitalar morreram em até 1 ano. Gheerbrant et al.^(3)^ também analisaram um banco de dados nacional e relataram 40% de mortalidade em 6 meses após a alta da UTI, com a presença de metástases independentemente e associadas a uma menor sobrevida. Os dois estudos anteriores incluíram apenas admissões não planejadas na UTI, enquanto o estudo de Puxty et al.^(1)^ incluiu admissões planejadas e não planejadas na UTI.

Os achados de Puxty et al.^(1)^ são importantes e representam um grande passo em direção a uma melhor compreensão dos pacientes com câncer que recebem alta para casa após uma doença grave; no entanto, duas perguntas permanecem sem resposta. A primeira pergunta é a seguinte: Todos os pacientes com câncer sofrem consequências negativas da internação na UTI? A segunda pergunta é: Quais são as causas das associações da internação na UTI com a progressão do câncer e a diminuição da sobrevida em longo prazo?

Provavelmente, pacientes com internação planejada de curta duração na UTI e poucas disfunções orgânicas são menos afetados pela internação na UTI, ao passo que pacientes com internação não planejada e longa na UTI e disfunções orgânicas múltiplas provavelmente são afetados e têm intervalo livre de progressão menos favorável do que pacientes com câncer, porém não internados na UTI. O estudo de Puxty et al.^(1)^ não nos deixa identificar claramente quais pacientes são afetados negativamente pela internação na UTI porque os dados não permitiram uma caracterização detalhada deles. Por exemplo, não sabemos a gravidade clínica dos pacientes na admissão ou sua evolução clínica durante a internação na UTI.

Duas hipóteses podem explicar as associações da admissão na UTI com a progressão do câncer e a diminuição da sobrevida em longo prazo. A primeira hipótese é a interrupção, o atraso ou a modificação do tratamento do câncer, pois as mudanças nessa terapêutica, mesmo com pequenos atrasos, estão associadas à menor sobrevida. A segunda hipótese é de que a imunossupressão prolongada após uma doença grave pode levar à progressão do câncer. A associação entre doença crítica e imunossupressão está bem estabelecida, e é possível que a imunossupressão induzida pela doença crítica possa causar a progressão do câncer devido às assinaturas imunossupressoras da doença crítica no microambiente do tumor, conforme resumido recentemente na sepse, que é uma causa comum de admissão na UTI em pacientes com câncer.^(4)^ Em apoio à ligação entre a imunossupressão e a progressão do câncer, estudo recente revelou aumento de 1 ano na incidência de câncer em sobreviventes de sepse em comparação com a população em geral.^(5)^ Se essas duas hipóteses forem confirmadas em estudos futuros, ou pelo menos uma delas, será possível intervir para reduzir a progressão do câncer e aumentar a sobrevida em pacientes com câncer após a internação na UTI. Uma possível intervenção é evitar a alteração do tratamento do câncer por meio da conscientização dos oncologistas para prescrever o tratamento anticâncer programado ou uma alternativa apropriada ao quadro clínico do paciente durante a hospitalização e do fornecimento de suporte clínico e socioeconômico para evitar o atraso ou a interrupção do tratamento do câncer após a alta hospitalar. Se for demonstrado que a imunossupressão prolongada após uma doença grave é relevante à progressão do câncer, outra intervenção poderia ser a introdução de terapias que aumentem a competência imunológica, como a imunoterapia.

Em conclusão, Puxty et al.^(1)^ confirmaram a associação entre a admissão na UTI e o intervalo livre de progressão menos favorável em pacientes com tumores sólidos. São necessários mais estudos para identificar quais pacientes são afetados negativamente pela internação na UTI e determinar as causas que associam a internação na UTI à progressão do câncer e à redução da mortalidade. Quando as causas forem conhecidas, poderemos implementar intervenções para diminuir a progressão do câncer e aumentar a sobrevida dos pacientes.
